# The role of MUC1 and MUC3 in the biology and prognosis of colorectal cancer

**DOI:** 10.1186/1477-7819-5-31

**Published:** 2007-03-09

**Authors:** Timothy J Duncan, Nicholas FS Watson, Ahmad H Al-Attar, John H Scholefield, Lindy G Durrant

**Affiliations:** 1 Academic Department of Clinical Oncology, Institute of Immunology, Infections and Immunity, University of Nottingham, City Hospital, Nottingham, UK; 2 Section of Gastrointestinal Surgery, Queen's Medical Centre, Nottingham, UK

## Abstract

**Background:**

MUC1 and MUC3 are from a large family of glycoproteins with an aberrant expression profile in various malignancies. Much interest has been focused on the role of these proteins in the development and progression of colorectal cancer; however, no previous studies have included the highly confounding variable of vascular invasion in their survival analysis. Using high throughput tissue microarray technology we assessed the prognostic value of MUC1 and MUC3 expression in the largest cohort of colorectal cancer patients to date. We propose that tumours lacking expression of MUC1 and MUC3 will be more likely to metastasise, due to previously observed loss of cell-cell adhesion, and this will therefore lead to more aggressive cancers with poorer prognosis.

**Methods:**

A tissue micro-array was prepared from tumour samples of 462 consecutive patients undergoing resection of a primary colorectal cancer. A comprehensive prospectively recorded data base with mean follow up of 75 months was collected and included common clinicopathological variables and disease specific survival. Immunohistochemical analysis of MUC1 and MUC3 expression was performed using antibodies NCL-MUC1 and 1143/B7 respectively, results were correlated with the variables within the database.

**Results:**

Positive expression of MUC1 and MUC3 was seen in 32% and 74% of tumours respectively. On univariate analysis no correlation was seen with either MUC1 or MUC3 and any of the clinicopathological variables including tumour grade and stage, vascular invasion and tumour type. Kaplan-Meier analysis demonstrated a significant reduction in disease specific survival with MUC1 positive tumours (p = 0.038), this was not seen with MUC3 (p = 0.552). On multivariate analysis, using Cox proportional hazards model, MUC1 expression was shown to be an independent marker of prognosis (HR 1.339, 95%CI 1.002–1.790, p = 0.048).

**Conclusion:**

MUC1 expression in colorectal cancer is an independent marker of poor prognosis, even when vascular invasion is included in the analysis. These results support previous studies suggesting a role for MUC1 in colorectal cancer development possibly through its effects on cell adhesion.

## Background

Colorectal cancer is the second most common cause of cancer related death in the developed world [1], in consequence advances in our understanding and treatment of colorectal cancers can potentially have a huge impact on cancer morbidity and mortality. Currently much of our understanding of cancer behaviour, including the prediction of likely patient outcomes, is based on histopathological parameters, and from this treatment is tailored to individual patients. At present TNM stage, tumour type and resection margin status are the most widely used parameters in planning adjuvant treatment. Tumour grade of differentiation, vascular invasion and more recently perineural invasion and tumour border configuration have also been used to assist the clinician in predicting colorectal tumour behaviour and hence subsequent patient management [2].

It is well recognised that clinical response and recurrence rates vary within the conventionally staged groups and that this reflects variation in the genetic and molecular make-up of these tumours. Molecular changes occur within cancer cells during tumour progression; these changes provide a potential insight into tumour development and metastasis.

Refining prognostic markers allow treatment to be more accurately tailored to individual patients, as well as suggesting potential mechanisms through which tumour progression occurs which in turn could provide targets for novel therapies.

MUC1 is a membrane bound glycoprotein which has been demonstrated to be predictive of tumour progression and worsening prognosis in both gastric [3-5] and colorectal cancer [6,7] including those related to HNPCC [8]. This increased expression has been seen more predominantly at the invasive tumour front [9].

MUC3 is also a trans-membrane glycoprotein which is seen in both colorectal cancers and normal colon [10]. Studies have shown an association between MUC3 expression and poor prognosis in a number of cancers including pancreatic [11], breast [12], gastric [13] and renal [14]. There is some evidence suggesting that MUC3 expression is reduced in colorectal cancers and that this varies between histological types [15]. The cellular distribution is also seen to be affected; apolar distribution is thought to reflect abnormal transport systems [16].

Whilst previous studies have suggested that tumour expression of MUC1 may be a useful prognostic factor in colorectal carcinoma [6,9] these studies have failed to include the presence or absence of vascular invasion in their analysis, this is known to be a highly significant prognostic factor in colorectal cancer[17]. We assessed the prognostic value of MUC1 on a larger set of colorectal tumours and included vascular invasion in our analysis to determine if MUC1 was truly independent as the previous studies have suggested. We also wanted to assess whether MUC3 demonstrated any prognostic influence on colorectal cancers as seen in other tumours.

Since its first description in 1998, tissue micro-array (TMA) analysis [18] has been employed for the immunohistochemical analysis of target protein expression in a wide range of primary tumour types. Initial fears that the reduced amount of individual tumour tissue analysed using this technique might not be representative of the tumour as a whole appear largely unfounded [19]. The strengths of this approach lie in its ability to provide a rapid turnover of results from very large patient cohorts, whilst reducing variability in experimental conditions and reducing costs [20]. Recently, in an attempt to overcome some of the reporting deficiencies inherent in prognostic tumour marker studies a set of guidelines, the reporting recommendations for tumour Marker prognostic studies (REMARK) have been proposed [21]. The reporting of this study therefore adheres to the REMARK guidelines. This TMA of colorectal cancer patients has previously been validated with a p53(-)/Bcl-2(+) phenotype, loss of HLA or over-expression of MICA all being independent markers of poor survival [22-24]. TMAs have also been utilised with MUC1 and MUC3 expression in breast cancer [12].

We have therefore used TMA technology to analyze expression of MUC1 and MUC3 in a series of 462 paraffin embedded colorectal tumour specimens, in conjunction with a detailed data base of clinicopathological variables including disease specific survival. We propose that tumours lacking expression of MUC1 and MUC3 will be more likely to metastasise, due to previously observed loss of cell-cell adhesion, and this will therefore lead to more aggressive cancers with poorer prognosis.

## Methods

### Patients and study design

The study population comprised a series of 462 consecutive patients undergoing elective surgical resection of a histologically proven sporadic primary colorectal cancer at the University Hospital, Nottingham, UK (table 1). These patients were treated between 1st January 1994 and 31st December 2000; this time period allowed meaningful assessment of the prognostic markers studied. All patients treated during this time-frame were considered eligible for inclusion in the study. Tumours were classified as mucinous carcinoma, when more than 50% of tumour volume consisted of mucin [25].

**Table 1:** Clinico-pathological variables for the patient cohort (n = 462)

**Gender**	Male	266 (58%)
	Female	196 (42%)
		
**Age (years)**	Median	72
	Range	57–89
		
**Status**	Alive	169 (37%)
	Dead (colorectal related)	228 (49%)
	Dead (non-colorectal related)	64 (14%)
	Unknown	1
		
**Tumour Grade**	Well differentiated	29 (6%)
	Moderately differentiated	353 (77%)
	Poorly differentiated	71 (15%)
	Unknown	8 (2%)
		
**Tumour Site**	Colon	238 (52%)
	Rectum	181 (39%)
	Unknown	43 (9%)
		
**TNM Stage**	0 (T_is_)	3 (1%)
	1	69 (15%)
	2	174 (38%)
	3	155 (33%)
	4	54 (12%)
	Unknown	7 (2%)
		
**Extramural Vascular Invasion**	Negative	224 (48%)
	Positive	128 (28%)
	Unknown	110 (24%)
		
**Histological type**	Adenocarcinoma	392 (85%)
	Mucinous carcinoma	51 (11%)
	Columnar carcinoma	4 (1%)
	Signet ring carcinoma	7 (1%)
	Unknown	8 (2%)

Only cases where the relevant pathological material was unavailable were excluded from the study. Follow-up was calculated from time of resection of the original tumour with all surviving cases being censored for data analysis at 31st December 2003, this produced a median follow up of 37 months (range 0–116) for all patients and 75 months (range 36–116) for survivors.

A prospectively maintained database was used to record relevant clinicopathological data, with data provided from the UK Office for National Statistics; this was available in more than 99% of cases. The information collected was independently validated through case note review of deceased patients. Disease specific survival was used as the primary end point; however, data was also collected on the various other relevant clinical and histopathological parameters these are summarised in table 1. There was no formal sample size calculation performed, although the inclusion of over 450 cases is in excess of most studies of prognostic tumour markers.

Adjuvant chemotherapy consisting of 5 FU and folinic acid was reserved for those patients with positive lymph nodes, although, surgical and adjuvant treatment was at the discretion of the supervising physician.

Prior ethical review of the study was conducted by the Nottingham Local Research and Ethics Committee, who granted approval for the study.

Construction of the array blocks incorporated a wide spectrum of electively resected colorectal tumours and was found to be broadly representative of the colorectal cancer population in the UK. 266 (58%) patients were male and 196 (42%) female. The median age at the time of surgery was 72 years, consistent with a median age at diagnosis of colorectal cancer of 70–74 years in the UK [26]. 69 (15%) tumours arrayed were TNM stage 1, 174 (38%) stage 2, 155 (34%) stage 3 and 54 (11%) stage 4; there were 3 cases of in-situ disease. These figures are comparable with national figures for distribution of stage 1–4 at diagnosis of 11, 35, 26 and 29% respectively [27]. The majority of tumours (392, 85%) were adenocarcinomas, and were most frequently of a moderate histological grade (353, 77%). 128 (28%) tumours were noted to have histological evidence of extramural vascular invasion, 224 (48%) had no evidence of vascular invasion, and this information was not available in 110 (24%) cases.

At the time of censoring for data analysis 228 (49%) patients had died from their disease, 64 (14%) were deceased from all other causes, and 169 (37%) were alive. The median five-year disease-specific survival for the cohort was 58 months, comparable with a national average of approximately 45% five-year survival for colorectal cancer in the UK [27].

### Specimen characteristics

All tumours received following resection in the operating theatre were incised, fixed immediately in 10% neutral buffered formalin followed by standard processing through to embedding in paraffin wax, ensuring optimal tissue fixation and preservation for histological examination.

Tissue micro-arrays were constructed as described previously [18]. For each tumour, 5 μm section slides stained with haematoxylin-eosin were first used to locate representative areas of viable tumour tissue. 0.6 mm needle core-biopsies from the corresponding areas on the paraffin-embedded tumour blocks were then placed at pre-specified coordinates in recipient paraffin array blocks using a manual tissue-arrayer (Beecher Instruments, Sun Prarie, WI). Array blocks were constructed with between 80–150 cores in each, with analysis of a single core from each case. Fresh 5 μm sections were obtained from each TMA block and placed on coated glass slides to allow the immunohistochemical procedures to be performed, preserving maximum tissue antigenicity.

### Immunohistochemistry

Immunohistochemical analysis of MUC1 and MUC3 expression was performed using a routine streptavidin-biotin peroxidase method. Tissue array sections were first deparaffinised with xylene, rehydrated through graded alcohol and immersed in methanol containing 0.3% hydrogen peroxide for 20 minutes to block endogenous peroxidase activity. In order to retrieve antigenicity, sections were immersed in 500 mls of pH 9.0 EDTA buffer and heated for 10 min in an 800 W microwave at high power, followed by 10 min at low power. Endogenous avidin/biotin binding was blocked using an avidin/biotin blocking kit (Vector Labs, USA). In order to block non-specific binding of the primary antibody all sections were then treated with 100 μl of 1/5 normal swine serum (NSS) in TBS for 15 min.

Test sections were incubated with 100 μl of mouse monoclonal antibodies recognising MUC 1 (NCL-MUC1 Novocastra, Newcastle, UK) which was found to show optimal staining at a dilution of 1/200 (v/v) in NSS/TBS, or MUC 3 (MUC3 1143/B7 NeoMarkers, California, USA) at 1/75 (v/v) NSS/TBS for 60 min at room temperature. Positive control tissue comprised whole sections of breast cancer tissue. The primary antibody was omitted from the negative control, which was left incubating in NSS.

After washing with TBS, all sections were incubated with 100 μl of biotinylated goat anti-mouse/rabbit immunoglobulin (Dako Ltd, Ely, UK) diluted 1:100 in NSS, for 30 min. Sections were washed again in TBS and next incubated with 100 μl of pre-formed streptavidin-biotin/horseradish peroxidase (HRP) complex (Dako Ltd, Ely, UK) for 60 min at room temperature. Subsequently, visualisation of MUC 1/MUC3 expression was achieved using 3, 3'-Diaminobenzidine tetra hydrochloride (DAB, Dako Ltd, Ely, UK). Finally, sections were lightly counterstained with haematoxylin (Dako Ltd, Ely, UK), dehydrated in alcohol, cleared in xylene (Genta Medica, York, UK) and mounted with distyrene, plasticiser and xylene (DPX – BDH, Poole, UK).

### Evaluation of MUC1 and MUC3 staining

The tumour cores were assessed by two observers (TJD and AHA) with regard to distribution and intensity of staining, both with extensive experience in the analysis of tissue micro-arrays. Tumours were classified according to a semi-quantitative system in a coded manner and blinded to the clinical and pathological parameters of the case. In the few cases (<5%) where there was discrepancy between the classification of cores a review, using a double headed microscope, was performed and a consensus reached.

For MUC1, tumours were scored according to the proportion of viable tumour cells within the tumour core which displayed unequivocal staining and scored: None = 0, <5% = 1, 5–29% = 2, 30–59% = 3, >60% = 4 in line with previous studies using the same antibody [6,28]. As the expression of MUC 3 was more uniform throughout positive tumour cores the intensity of staining was used as the discriminator with tumours categorised as showing negative, low, moderate and high intensity of MUC3 expression. For the purposes of survival analysis tumours were further categorised as either negative or positive for marker expression. Tumours were considered positive for MUC 1 expression when at least 30% of cells demonstrated positive staining, this is in keeping with previous studies [6,28]. For MUC3 tumours displaying moderate or high intensity staining were considered positive, with the remainder considered negative

### Statistical analysis

Statistical analysis of the study data was performed using the SPSS package (version 14 for Windows, SPSS Inc., Chicago, IL). Pearson χ^2 ^chi-square tests were used to determine the significance of associations between categorical variables. Disease-specific survival calculations included all patients whose death related to colorectal cancer. In contrast, patients whose deaths resulted from non-colorectal cancer related causes were censored at the time of death. Kaplan-Meier curves were used to assess factors which influenced survival. The statistical significance of differences in disease-specific survival between groups with differing MUC1 and MUC3 expression was estimated using the log-rank test. The Cox proportional-hazards model was used for multivariate analysis in order to determine the relative risk and independent significance of individual factors. In all cases p-values < 0.05 were considered as statistically significant.

## Results

### Patient and histopathological variables and prognosis

Univariate relationships between known patient/tumour characteristics and DSS were evaluated using the log-rank test (see Table 2). There appeared to be no significant differences in DSS between patients of either gender. Similarly when patient age was considered in three groups (patients 64 years or younger at the time of surgery, patients 65–79 years, and those 80 years and over), no significant differences in DSS were noted. The site of tumour i.e. colon or rectum had no influence on DSS.

**Table 2:** Univariate survival analysis of patient/tumour characteristics

Variable	Total Number (%)	Mean DSS (months)	95% CI (months)	*log rank *p value
**Gender**				
Male	266 (57)	64	57–70	
Female	196 (42)	64	57–71	0.8374
**Age (years)**				
<64	71 (15)	66	54–77	
65–79	316 (68)	64	58–69	
80+	75 (16)	61	49–72	0.9602
**Tumour Grade**				
Well	29 (6)	66	45–86	
Moderate	353 (76)	65	60–71	
Poor	71 (15)	57	46–69	
Unknown	9 (2)	52	7–37	0.0627
**Tumour Site**				
Colon	238 (52)	66	59–72	
Rectum	181 (39)	65	58–73	
Unknown	43 (9)	46	31–60	0.0620
**TNM Stage**				
0/I	72 (15)	98	89–106	
II	174 (38)	79	71–86	
III	155 (33)	52	44–59	
IV	54 (12)	8	6–10	
Unknown	7 (2)	29	8–50	<0.0001
**Vascular Invasion Status**				
Absent	224 (48)	75	68–81	
Present	128 (28)	38	31–45	
Unknown	110 (24)	68	59–78	<0.0001
**Tumour type**				
Adenocarcinoma	392 (84)	65	60–70	
Mucinous	51 (11)	66	52–79	
Columnar	4 (1)	51	8–94	
Signet ring	7 (2)	27	11–44	0.4860
**MUC1 expression**				
High	127 (32)	54	45–62	
Low	276 (68)	65	60–71	0.038
**MUC3 expression**				
High	286 (74)	63	57–69	
Low	101 (26)	59	49–69	0.552

Tumour grade showed a trend towards reduced survival with increasing dedifferentiation, with colorectal cancer related deaths occurring in 34.5% (10/29), 48.4% (171/353) and 56.3% (40/71) of patients with well, moderately and poorly differentiated tumours respectively, although this did not reach statistical significance in our cohort of patients. The majority of tumours were adenocarcinomas, however non-adenocarcinoma tumours, did not have a statistically significantly poorer prognosis in this series. Extramural vascular invasion had a strong correlation with survival, 72% (92/128) of patients with evidence of vascular invasion died from colorectal cancer related causes, compared with only 39% (87/224) in patients without. In cases where the vascular invasion status was unknown an intermediate mean DSS was noted. The association between vascular invasion and DSS was highly significant and log-rank testing (log-rank = 44.30, p < 0.0001).

The strongest association of clinicopathological variables with DSS was seen with TNM staging (log-rank = 211.37, p < 0.0001), showing a progressive reduction in DSS with increasing tumour stage.

### Tumour marker expression

#### MUC1

Analysis of MUC1 expression was possible in 403 of the 462 tumours on the TMA (87%), with the remainder being lost during antigen retrieval or not demonstrating viable tumour cells within the core. This level of core loss is within the rates described by previous authors using TMAs [29,30]. The majority of staining was seen within the cytoplasm and cell membrane, no staining was seen within the nucleus or surrounding stromal tissue. No staining was seen in 188 (47%) tumours, with <5% and 5–30% of cells staining in 47 (12%) and 41 (10%) respectively. There was 30–60% staining in 62 (15%) cores and greater than 60% in 65 (16%). When dividing the tumours according to previous studies [6,28] 276 (68%) tumours were MUC1 negative and 127 (32%) positive.

#### MUC3

Analysis of MUC3 expression was possible in 387 cores (84%). The staining was seen mainly within the cytoplasm 354 (91%) but also with the cell membrane 147 (38%), there was no nuclear staining but occasional stromal staining seen. The majority of tumours displayed either moderate 187 (48%) or strong staining 99 (26%), weak or no staining was seen in 68 (18%) and 33 (8%) respectively.

**Figure 1:**
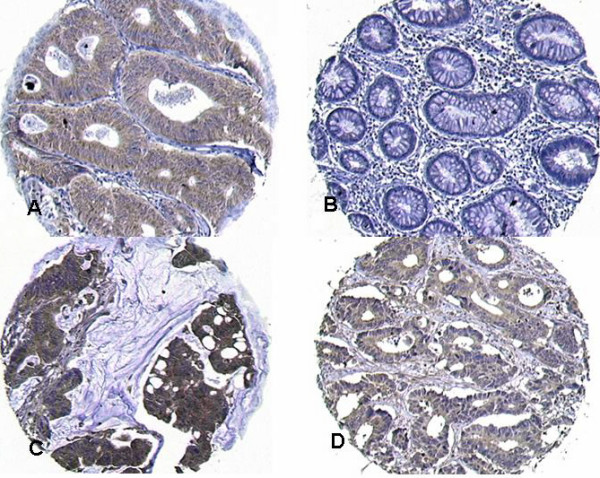
**Immunohistochemical staining of tissue microarray cores with MUC1 and MUC3 antibodies**. A & B show cores from tumour demonstrating positive (A) and negative (B) MUC1 staining. C & D show cores of tumour demonstrating strong (C) and weak MUC 3 staining. All are at × 100 magnification.

Representative examples of positive and negative staining for each antigen are shown in figure 1.

### Relationships between tumour markers and standard clinicopathological variables

#### MUC1

For the purposes of analysis the tumours were divided into those with positive or negative expression, as described previously [6,28]. There did not appear to be any relationship between any of the clinicopathological variables, including stage, and MUC1 expression (see table 3).

**Table 3:** Patient and Tumour characteristics in relation to MUC1 expression (n = 403)

Variable	Total Number	Number (%) MUC1 +	Number (%) MUC1 -	χ^2 ^test (p value)
**Gender**				
Male	227	73 (32)	154 (68)	
Female	176	54 (31)	122 (69)	0.418
**Tumour Grade**				
Well	27	11 (41)	16 (59)	
Moderate	302	94 (31)	208 (69)	
Poor	65	18 (28)	47 (72)	
Unknown	9	4 (44)	5 (56)	0.527
**Tumour Type**				
Adenocarcinoma	344	111 (32)	233 (68)	
Mucinous	42	10 (24)	32 (76)	
Other	9	2 (22)	7 (78)	
Unknown	8	4 (50)	4 (50)	0.412
**Tumour Site**				
Colon	211	69 (33)	142 (67)	
Rectum	155	43 (28)	112 (72)	
Unknown	37	15 (40)	22 (60)	0.279
**TNM Stage**				
0	3	0	3 (100)	
I	59	19 (32)	40 (68)	
II	152	44 (29)	108 (71)	
III	134	43 (32)	91 (68)	
IV	48	17 (35)	31 (65)	
Unknown	7	4 (57)	3 (43)	0.501
**Vascular Invasion Status**				
Positive	116	38 (33)	78 (67)	
Negative	193	67 (35)	176 (65)	
Unknown	94	22 (23)	72 (77)	0.145

#### MUC3

As the majority of tumour cells within each core expressed a uniform staining pattern, the cores were classified according to intensity of staining as opposed to the proportion of cells staining. Cores were deemed positive if moderate or strong staining was seen. Using this system 286 (74%) tumours were positive and 101 (26%) negative. No correlation between MUC3 cytoplasmic expression and any clinicopathological variables, including stage, was seen (see table 4). Equally there was no correlation of membranous staining with any clinicopathological variables (data not shown).

**Table 4:** Patient and Tumour characteristics in relation to MUC3 expression (n = 387)

Variable	Total Number	Number (%) MUC3 +	Number (%) MUC3 -	χ^2 ^test (p value)
**Gender**				
Male	221	167 (76)	54 (24)	
Female	166	119 (72)	47 (28)	0.390
**Tumour Grade**				
Well	22	20 (91)	2 (9)	
Moderate	295	227 (77)	68 (23)	
Poor	61	31 (50)	30 (50)	
Unknown	9	8 (89)	1 (11)	0.389
**Tumour Type**				
Adenocarcinoma	326	245 (75)	81 (25)	
Mucinous	43	30 (70)	13 (30)	
Other	10	4 (40)	6 (60)	
Unknown	8	7 (87)	1 (13)	0.061
**Tumour Site**				
Colon	201	142 (71)	59 (29)	
Rectum	153	116 (76)	37 (24)	
Unknown	33	28 (85)	5 (15)	0.160
**TNM Stage**				
0	3	2 (67)	1 (33)	
I	58	47 (81)	11 (19)	
II	146	112 (77)	34 (23)	
III	128	89 (70)	39 (30)	
IV	45	30 (67)	15 (33)	
Unknown	7	6 (86)	1 (14)	0.391
**Vascular Invasion Status**				
Positive	106	78 (73)	28 (27)	
Negative	194	149 (77)	45 (23)	
Unknown	87	59 (23)	28 (32)	0.283

### Relationship between tumour markers and patient survival

**Figure 2:**
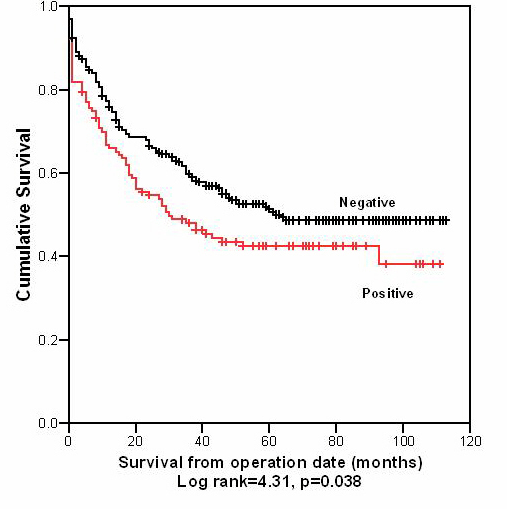
Kaplan-Meier plot for disease specific survival, MUC1 (+) vs. MUC1 (-) tumours (n = 403).

**Figure 3:**
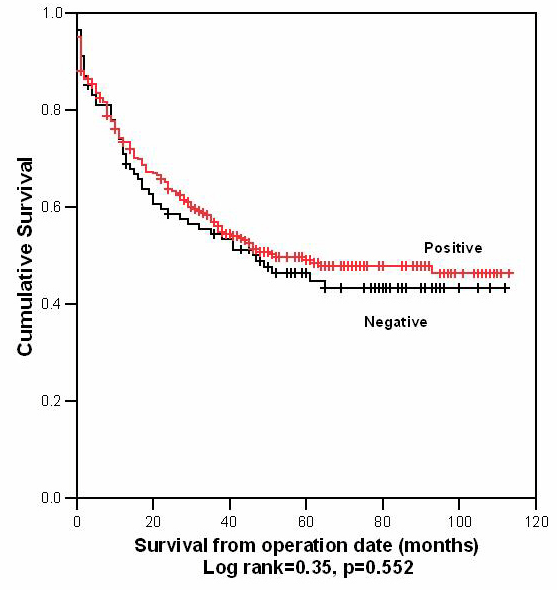
Kaplan-Meier plot for disease specific survival, MUC3 (+) vs. MUC3 (-) tumours (n = 387).

Correlation between MUC1 and MUC3 expression and DSS was assessed using Kaplan-Meier plots and log rank testing (see table 2, figures 2 and 3). A significant association was seen between tumours with high MUC1 expression and a reduced DSS (mean DSS 54 months vs. 65 months; p = 0.038). In contrast, there was no correlation between MUC3 expression and DSS.

In order to determine the relative influence of MUC1 and other patient and tumour variables known to affect prognosis, a multivariate analysis was performed using the Cox proportional hazards model. We included only those variables which had been shown to be significantly related to DSS on univariate analysis i.e. intramural vascular invasion and TNM stage (see table 5). In this model, vascular invasion (p < 0.001) and TNM staging (p < 0.001) were seen to retain independent prognostic significance. High expression of MUC1 was also seen to be an independent prognostic marker of poor outcome, with a hazard ratio of 1.339 (95%CI 1.002–1.790, p = 0.048), when compared with tumour demonstrating low MUC1 expression.

**Table 5:** Cox multivariate regression analysis of variables in relation to disease specific survival

Variable	Hazard Ratio	95% CI	p value
**TNM Stage**			
0–II	1		
III–IV	2.386	2.074–3.878	<0.001
**Vascular Invasion Status**			
Negative	1		
Positive	1.830	1.314–2.548	
Unknown	1.392	0.914–1.933	<0.001
**MUC1 expression**			
Low	1		
High	1.339	1.002–1.790	0.048

## Discussion

This study investigates the role of MUC1 and MUC3 as prognostic markers in colorectal cancer. Previous studies have suggested a link between MUC1 and MUC3 expression and poor prognosis both in colorectal and other tumour types [3-8]. These studies have frequently suffered from small sample sizes and/or heterogeneous methodology and study populations. The current study comprises the largest analysis of MUC1 and MUC3 expression in colorectal cancer to date; including 463 consecutively treated representative patients, who were representative of the colorectal cancer population within the UK. With a comprehensive data set of clinicopathological variables and patient outcome, over a median 3 year postoperative period, a thorough and comprehensive analysis was possible between these variables and disease specific survival.

In our study population 32% of tumours were positive for MUC1. This compares favourably with previous authors work, who also used the same semi-quantitative scoring system and found 32% and 43% MUC1 positivity in colorectal tumours respectively[6,28].

In our study population MUC1 expression was not related to any of the clinicopathological variables examined. Some previous studies demonstrated increased MUC1 expression was related to increasing TNM or Dukes stage [31-33]; however, a number of other studies are in line with our findings [9,34]. Variations in the findings of the current and previous studies may relate to differences in immunohistochemical protocols, antibodies used, scoring systems and area of the tumour examined e.g. Hiraga *et al *and Kimura *et al *only assessed staining at the invasion front [31,32]. A large study by Lugli *et al *examines the prognostic significance of MUC1 and MUC2 in relation to differing mismatch repair status in colorectal cancer, with tumours divided into three subgroups. Significant correlations were found in the "mismatch repair proficient group" between MUC1 positivity and tumour stage and grade [33]. There was no such correlation in our cohort, however our analysis did not involve sub-stratification of the population and hence may explain the dissimilar results.

Univariate and multivariate analysis of our patient population confirmed that TNM staging and vascular invasion are strong independent prognostic markers in colorectal cancer. Of particular interest was the large effect vascular invasion had on survival. Presence of vascular invasion reduced mean DSS significantly (38 vs 75 months p < 0.0001), yet no previous studies investigating the prognostic value of MUC1 have included this obviously strong predictor of survival in their analysis. Our data confirm that high expression of MUC1 in colorectal cancer confers a worse prognosis both on univariate and multivariate analysis, even when taking into account the potentially confounding influence of vascular invasion status.

The association of MUC1 with poor prognosis has been linked to effects on cell adhesion and the potential for metastasis. Regimbald *et al *[35] showed that MUC1 was a ligand for ICAM-1 in breast cancer and might have a pivotal role in haematogenous spread, and it has been speculated that this mechanism may occur in colorectal cancer [36]. MUC1 is also seen to have effects on the extra cellular matrix components through inhibition of kalinin and laminin [37,38].

MUC1 has been demonstrated to affect beta-catenin, a nuclear transcription factor, and its intracellular distribution has been shown to influence progression of colorectal cancer [39], it has been suggested that MUC1 exerts some of it's effects through interaction with beta-catenin, with over expression of MUC1 leading to increased levels of nuclear beta-catenin [40]. A recent study has shown that the co-expression of MUC1 and nuclear beta-catenin at the invasion front of colorectal tumours may be correlated with a worse prognosis [9].

MUC3 expression was present in moderate to high levels in 76% of tumours assessed. Some studies have suggested that MUC3 may in fact be down-regulated in colorectal cancer compared with normal colon [10,15]. We did not see any correlation between the clinicopathological variables and MUC3; in particular there was no correlation with tumour stage as is seen with gastric cancers [13]. Furthermore, MUC3 expression did not appear to correlate with prognosis, as has been reported in other tumour types [11-14]. Rakha *et al *demonstrated MUC3 expression in 91% of breast cancers which was associated with increased local recurrence and lymph node stage. They argued that membranous expression of MUC3 was a poor prognostic feature, which correlated with higher grade and poorer Nottingham Prognostic Index (NPI) [12]. Wang reported that increased MUC3 expression in gastric cancer worsened prognosis, with no significant differences in expression seen in relation to patient sex, tumour location, grade of differentiation, serosal invasion, or Lauren's type. However MUC3 expression was higher in those with metastasis (p < 0.01) and in clinical stage III–IV disease compared to I–II (p < 0.05). MUC3 were not detected in the normal gastric mucosa [13]. MUC3 showed a progressive increase in expression with pancreatic intraepithelial neoplasia of increasing dysplasia and was also highly expressed in ductal adenocarcinoma [11].

Normal lung tissues exhibited a distinct pattern of mucin gene expression, with high levels of MUC1 and low levels of MUC3 immunoreactivity and mRNA. In contrast, lung adenocarcinomas, especially well-differentiated cancers, exhibited increased MUC1 and MUC3 mRNA levels [41]. Copin *et al *found that coexpression of MUC3 and MUC1 was constant among lung adenocarcinomas [42].

## Conclusion

We have demonstrated that using TMA technology and a large cohort of colorectal cancer patients with robust long term follow up data that biomarkers of prognosis can be reliably assessed. Our data clearly demonstrates a role for MUC1 in the progression of colorectal cancer, probably through its effects on cell adhesion and metastasis. MUC1 expression appears to function as an independent prognostic marker in colorectal cancer even when the conventional variables of tumour stage and vascular invasion status are included in the analysis.

## Competing interests

The author(s) declare that they have no competing interests.

## Authors' contributions

**TJD**: Performed IHC, analyzed data and wrote manuscript

**NFSW**: Contributed to study design and interpretation of results

**AHA**: Analyzed data and interpretation of results

**JHS**: Conceived study and participated in its design

**LGD**: Conceived study, participated in its design and interpretation of results
